# Recent Advances in Solid-State Modification for Thermoplastic Polymers: A Comprehensive Review

**DOI:** 10.3390/molecules29030667

**Published:** 2024-01-31

**Authors:** Jonas José Perez Bravo, Carolane Gerbehaye, Jean-Marie Raquez, Rosica Mincheva

**Affiliations:** Laboratory of Polymeric and Composite Materials, CIRMAP, University of Mons, 23, Place du Parc, 7000 Mons, Belgium; jonasjose.perezbravo@umons.ac.be (J.J.P.B.);

**Keywords:** solid state modification, thermoplastic polymers, recycling, upcycling

## Abstract

This review introduces groundbreaking insights in polymer science, specifically spotlighting a novel review of the solid-state modification (SSM) approach of thermoplastic polymers, a method not extensively explored. Unlike traditional melt polymer modification, SSM stands out by incorporating monomers or oligomers into the amorphous phase of polymers through innovative exchange reactions. The background of the study places thermoplastics within the context of their increased use over the past century, highlighting their versatility in various applications and the associated environmental and health concerns due to certain additives. The results section outlines the unique aspects of SSM and its increasing recognition for its potential to enhance material performance in areas such as catalysts and composites. It also discusses the application of SSM in modifying different thermoplastic polymers, highlighting various studies demonstrating the method’s effectiveness in altering polymer properties. Finally, this work emphasizes SSM’s importance in environmental sustainability and its potential in the recycling and upcycling of plastic materials. It acknowledges the challenges and future perspectives in the field, particularly regarding the scalability of SSM techniques for industrial applications and their role in advancing a circular economy in the polymer industry.

## 1. Introduction

Have you ever considered the amazing journey of plastics from being a novel invention to a global environmental concern? In the past century, the use of plastic materials has increased significantly in various applications, from single-use items to engineering-grade polymers (e.g., food preservation, healthcare, transportation, and energy), thanks to their extensive range of properties such as permeability, waterproofing, hydrophobicity or hydrophilicity, stiffness, or flexibility. Plastic production has grown to an estimated annual production of over 390 million tons in 2021, with a growth rate of 4% per year [1].

What gives them such diverse properties as permeability, waterproofing, and flexibility? It is a blend of polymeric co-partners and other additives. But here is the catch: while these additives optimize properties, they can also lead to mechanical weaknesses, compatibility issues, and even environmental and health hazards due to toxic substances like phthalate plasticizers and brominated flame retardants [2,3,4]. These additives have been found to cause various health issues, including endocrine disruption and reproductive problems. These harmful substances can seep out of products and accumulate in the environment, putting both wildlife and humans at risk. Additionally, brominated flame retardants, which are used to reduce the flammability of materials, have been linked to neurodevelopmental issues and are highly persistent in the environment, leading to long-term ecological consequences [5,6]. Therefore, plastic materials are often associated with various additives, such as polymeric co-partners, to achieve specific properties [7,8,9]. These adjustments can improve processability, reduce costs, or extend the material’s lifespan. However, when added in excess, certain additives can significantly affect the final material’s mechanical properties and cause compatibility issues within the matrix [10]. It is crucial to maintain a good balance between the additive’s loading and its impact on the initial properties of the material, which often requires a compatibilization step. Additionally, some additives are considered to be controversial due to their toxicity during fabrication and use of plastic material. This can have harmful effects on the environment and human health, as certain compounds can migrate out of plastics [11,12,13]. Examples of such additives include phthalate plasticizers and brominated flame retardants [14,15,16]. On the other hand, the global plastic waste crisis requires urgent action from science and industry to manage plastic waste through reuse, recycling, or upcycling [17]. These methodologies involve modifying thermoplastic polymers, including polymers used during the melting stage, which requires high temperatures. However, these processes can produce undesirable changes in the properties of plastics due to various factors, including thermal and oxidative degradation, as well as microstructural changes [18,19].

Therefore, there is a growing interest in developing efficient techniques to modify thermoplastic materials by adding desired functionalities using low-content additives and avoiding detrimental reactions during their processing [20,21]. Such a technique relies on the solid-state modification (SSM) of polymeric materials, which exploits the mobility of the amorphous phase within semi-crystalline polymers [22]. This method involves using mechanical forces to drive through the incorporation of monomers or oligomers into the amorphous phase of the polymer through multiple exchange reactions [23]. The temperature plays a critical role during this process as it needs to be above the Tg of the semi-crystalline polymer and close to its Tm. The exchange reactions mainly occur in the amorphous phase, which means that the main crystalline phase and its associated properties remain unaffected. This technique can be considered a post-synthetic modification method that allows for the preservation and/or modulation of desired properties.

But what makes SSM unique, and why is it not widely known despite its apparent advantages? With only 21 publications on the topic over three decades, SSM remains a niche yet promising field. It holds the key to adding functionalities to thermoplastics with minimal additives and avoiding adverse reactions during processing. SSM is increasingly recognized for its potential to enhance material performance from catalysts to composites. These developments have covered some polymers, including thermoplastic and thermosets. The main purpose of these investigations was to modify inherent properties, particularly in the case of composites. Based on the SSM references histogram, the years of high publication activity belong to the period between 2012 and 2022. However, SSM has continued to investigate the topic, presenting an increasing number of articles as the years go by, especially after 2012. Additionally, SSM has expanded to different contemporary peaks research issues, such as catalysts and composites, often serving as a tool to investigate compositional effects and materials behavior, as well as to improve end-product performance.

In this respect, this review will focus on describing the current perspective of using pre-formed thermoplastic polymers with SSM methodologies to modify thermoplastic polymers with desired functionalities. By contrast with solid-state polymerization (SSP), which relies on a kind of bulk polymerization technique, SSM goes beyond by encompassing the concept of thermo-mechanochemistry. Thermo-mechanochemistry is a highly specialized field of chemistry that delves into the interaction between thermal (heat), mechanical (force or pressure), and chemical factors in material reactions and transformations [24,25]. This study area is particularly significant for understanding and designing materials that undergo chemical changes due to mechanical stress or thermal conditions. It combines the principles of thermodynamics, mechanical engineering, and chemistry to analyze and manipulate the behavior of materials. Finally, the aim is to provide the reader with information not only to understand the relevant solid-state modification technique but also to encourage future research, as we believe that SSM methodologies are a continuously thrilling field.

## 2. Thermo-Mechanochemistry: A Scientific Field That Can Be Focused on Solid-State Modification through Mechanical Means

**Figure 1:**
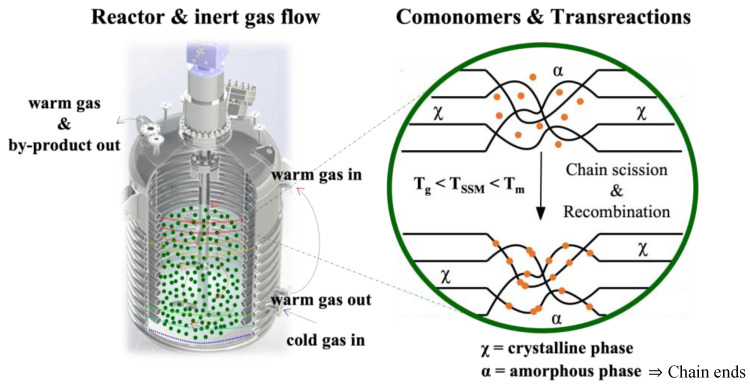
Schematic representation of solid-state modification.

SSM is a technique that has not been widely explored in the literature, despite being at the forefront of thermo-mechanochemistry. It involves modifying polymeric matrices after synthesis to extend their range of applications or improve a specific targeted property. This technique is related to solid-state polymerization, which increases the molecular weight of semi-crystalline polycondensates. SSP is commonly used in the production of glass fiber-reinforced PET grades [26]. This technique enables the formation of copolymers without requiring a high-temperature melt polycondensation reaction. This process demands the precise control of reaction parameters and significant thermal stability. SSM involves heating the starting material in an inert atmosphere or vacuum to a temperature below its melting point. This temperature is high enough to initiate and propagate the exchange reaction in the presence of a comonomer. However, the material must be above its glass transition temperature to ensure enough mobility of the end groups in the amorphous phase. It is also essential to prevent the crystalline phase from participating in the reaction. The image of Figure 1 illustrates this basic principle [22].

**Figure 2:**
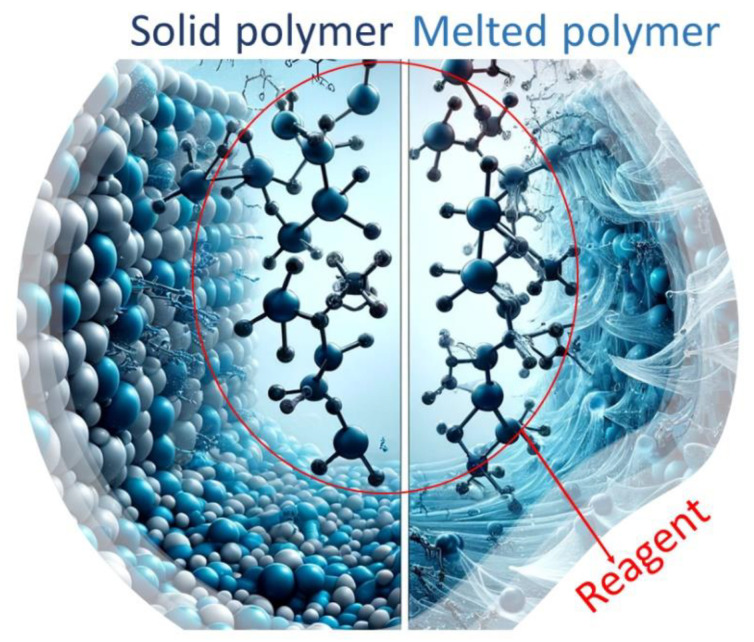
Schematic representation of how the same reagent can modify a polymer both in its melted and solid-state forms.

The reaction mechanisms involved in SSM are similar to those in melt modification (Figure 2) [23]. However, a solid-state matrix is utilized as the reaction medium instead of a melt polymeric matrix. It is important to note that SSM has limitations on end-group diffusion due to restricted mobility. This is not an issue in melt or dissolution technology. These limitations become more severe over long reaction times when nearby functional groups already react. As a result, the concentration and distribution of these groups are reduced locally, making the migration of unreactive chain ends crucial for the reaction to continue. Removing condensate effectively through diffusion is crucial for achieving high SSM rates and extents. Although internal and surface diffusion are theoretically different, they are related and influenced by similar parameters. This is because both internal and surface diffusion eliminates concentration gradients of by-products in the reaction zone. This eliminates depolymerization and promotes a shift in the reaction equilibrium towards the desired products [22].

In SSM processes, the removal of by-products is accomplished by either applying a vacuum or through the convection caused by an inert gas at atmospheric pressure. The oligomers that are formed during the reaction can sublimate into the gas phase along with the condensate. This process can also involve heating the mixture under a continuous flow of inert gas (open system), which promotes the removal of by-products. During SSM (Solid State Polymerization) processes, the elimination of by-products is achieved either by using a vacuum or the convection created by an inert gas at atmospheric pressure. The oligomers that are produced during the reaction can vaporize into the gas phase alongside the condensate. The primary objectives of using an inert gas are to remove the condensate, prevent polymer oxidation by excluding oxygen from the reactor atmosphere, and to heat the reaction mixture. The most used inert gases include nitrogen, carbon dioxide, helium, superheated steam, and supercritical carbon dioxide. This process can also involve heating the mixture under a continuous flow of inert gas (open system), which helps to remove by-products.

## 3. Parameters That Affect the Solid-State Modification Technique

Solid-state modification is a complex process, and several parameters can be controlled to attain the desired polymer material. These parameters include temperature, gas flow, vacuum applied, crystallinity, catalysts, and others. The factors that impact the SSM technique are listed below [23].

### 3.1. Temperature

**Figure 3:**
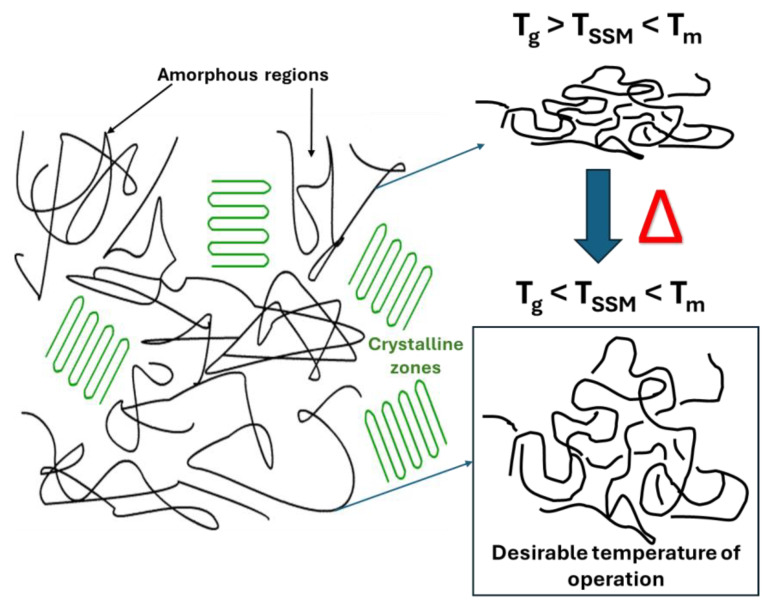
Schematic representation of desirable temperature to carry out the SSM.

The temperature at which the SSM process occurs is a crucial factor that impacts almost all other process stages [22]. The reaction temperature mainly affects the reaction kinetics [27]. Maintaining a temperature range optimal for maximizing the reaction rate while avoiding undesired reactions such as partial melting, sticking, or cyclization is crucial. However, if the monomer has a high melting temperature, the temperature range can be broader, and the impact of temperature on the process rate is minimized. It is not feasible to carry out SSM on polymers with a melting temperature that is too low [28]. Raising the temperature of a process quickens its overall rate, the chemical reaction, and the movement of terminal functional groups and mass transport [22,27,28]. However, the temperature should not be too close to the melting temperature to avoid particle agglomeration. In summary, the reaction should be conducted above the glass transition temperature and between 10 °C to 40 °C below the melting temperature of the crystalline phase of the prepolymer (Figure 3) to prevent particle agglomeration [26,27,29].

### 3.2. Atmosphere

SSM can be carried out using either a vacuum or an inert gas, such as nitrogen. The main objectives of these reaction conditions are to remove by-products, inhibit polymer oxidation by excluding oxygen from the reactor atmosphere, and heat the reaction mass (in the case of gas flow only). The difference between these two methods is that for vacuum, the rapid removal of by-products is affected by the applied pressure, while for gas flow, it depends on the flow rate of the inert gas used. In SSM processes, the commonly used inert gases include nitrogen, carbon dioxide, helium, and superheated steam [30]. When performing the solid-state modification process, it is important to take into account the characteristics of the inert gas being used, since they may have an impact on the process. SSM can be carried out in two different ways: either under a continuous flow of inert gas, which is referred to as an open system and is primarily focused on removing by-products, or under a stagnant inert gas atmosphere, which is known as a closed system and helps to limit the loss of monomers and oligomers [31,32].

### 3.3. Crystallinity

**Figure 4:**
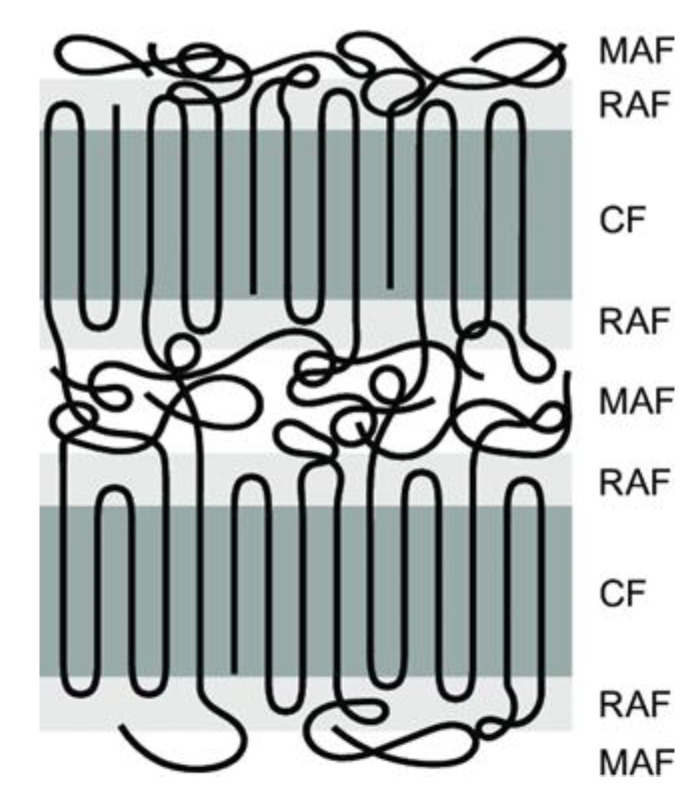
Schematic representation of three-phase model, i.e., mobile amorphous fraction (MAF), the rigid amorphous fraction (RAF) and the crystalline fraction (CF).

Crystallinity plays a crucial role in SSM as it impacts the movement of chain end groups and the diffusion of by-products. The effect of crystallinity on the rate of SSM is two-fold. Higher crystallinity levels result in an increased concentration of end groups released into the amorphous phase, which in turn leads to an acceleration of the reaction rate [26]. The polymer chains become less mobile as the reaction progresses due to increased crystallinity. This decrease in mobility slows down the rate of SSM. When reactions are limited by by-product diffusion, high crystallinity also reduces the rate of SSM. However, in processes controlled by chemical reactions, high crystallinity actually increases the rate of SSM due to the concentration of end groups in the amorphous phase [33]. In order to prevent particles from sticking together, it is necessary for them to have a high level of crystallinity. In particle agglomeration, particles must have sufficiently high crystallinity [34]. Furthermore, it has been shown for many years that semi-crystalline polymers can be described using a three-phase model. This means that they contain a crystalline phase and an amorphous phase. However, the amorphous phase is divided into two zones: a mobile and a rigid amorphous zone, as shown in Figure 4 [35]. The exchange reactions should occur in the more mobile zones that can better tolerate changes [32].

### 3.4. Catalysts

Catalysts are crucial for the rate of polymerization and modification reactions. Their role extends beyond simply accelerating reactions. They also contribute to the quality and properties of the final polymer product by influencing the chain initiation and growth, as well as the interactions among the polymer constituents. Additionally, catalysts can lead to complete conversions of specific groups, significantly impacting the polymerization rate, as observed with phase-transfer catalysts [36]. This ability to control reaction rates is crucial, especially in processes where removing by-products like water is necessary for the desired reaction outcomes [22]. The incorporation of catalysts into the polymerization process can be done at different stages. They can be added during the production of the prepolymer or while melting the prepolymer, providing flexibility in controlling the polymerization process. This versatility allows for a tailored approach to achieve specific properties and characteristics in the final thermoplastic polymer product [31].

## 4. Thermoplastic Polymers Modified through Solid-State Modification Technique

**Figure 5:**
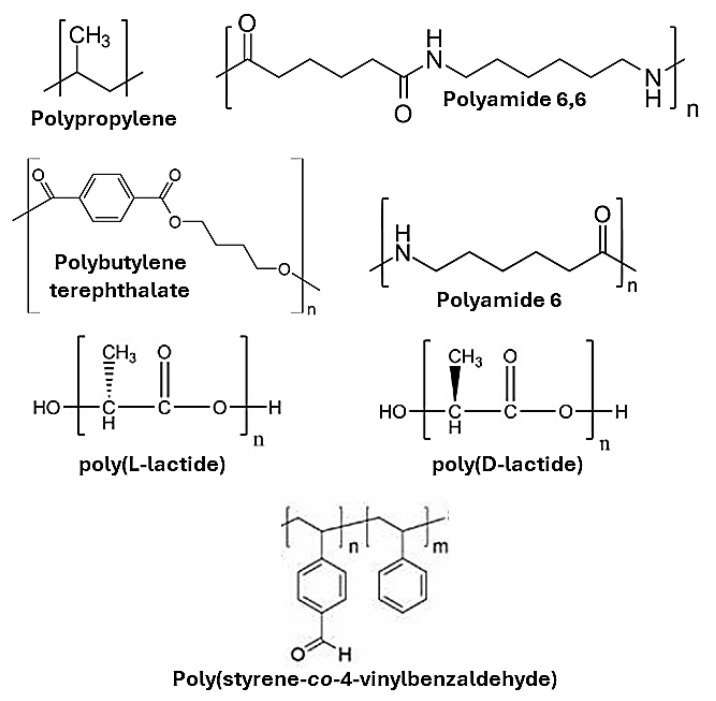
The structure of thermoplastic polymers that have been modified by solid-state.

**Figure 6:**
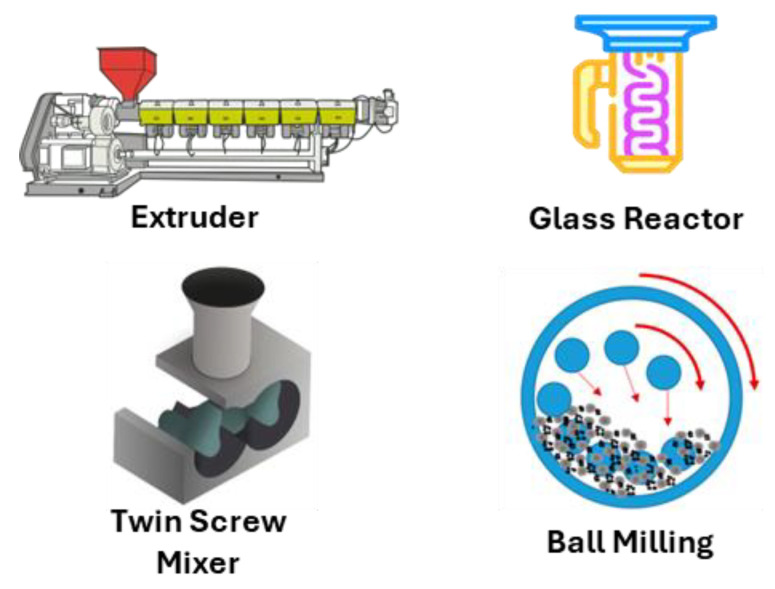
Instruments have been employed to modify thermoplastic polymers in the solid state.

Solid-state modification of post-synthetic thermoplastic polymers is a rapidly evolving technique with numerous advantages and potential applications. This approach is particularly attractive because it allows adding new functionalities to existing polymer matrices, thereby extending their utility or assigning new purposes [37]. One of the main benefits of SSM is its ability to overcome solubility issues often encountered with certain polymers. Additionally, it offers a more environmentally friendly alternative to traditional methods, featuring greater selectivity and the potential for scalability [38]. SSM in thermoplastic polymers is cost-effective and allows for more significant creative variability and reduced degradation reactions compared to solutions or melts. It has been applied effectively to reprocess and recycle condensation plastics, enhancing their properties without needing solid-state re-polymerization. This makes high-end recycling economically attractive, especially for high-value-added engineering applications like manufacturing higher-performance fibers and films with high tensile properties [39]. SSM is especially beneficial due to its compatibility with a wider range of monomers, including those that are fragile or have a low degradation temperature, thanks to the milder conditions it employs, particularly in terms of temperature control (Figure 5). This expands the scope of modifications that can be made to thermoplastic polymers. Several research groups are exploring various modifications of polymers using SSM techniques (Figure 6). This includes mechanochemistry, which allows for the physical blending and alteration of polymer structures without the need for solvents or high temperatures, as typically required in other polymer processing methods. However, it is important to note that, to date, only a few thermoplastic polymers have been effectively modified using these techniques. Table 1 summarizes the modified thermoplastic polymers, equipment, and important input information.

**Table 1:** Summarized information of thermoplastic polymer’s physicals proprieties, as well as inputs and instruments to carry out SSM.

Thermoplastic Polymer	T_m_ (°C)	T_g_ (°C)	T_SSM_ (°C)	Equipment’s to Carry out SSM	Co-Monomers	Reference
Polypropylene	NR	NR	75–85	Ball-milling process	maleic anhydride	[40,41]
Polyethylene	NR	NR	75–85	Ball-milling process	maleic anhydride	[40]
Polypropylene	NR	NR	60	Glass reactor	Silica	[42]
Poly(butylene terephthalate)	225	40–45	160–175	Glass reactor	1,4BD, Manx, Glux, Galx and FADD	[43,44,45,46,47]
Poly(butylene terephthalate)	225	45	190	Twin screw extruder	DDO	[17]
Polyamide-6	221	NR	180	Glass reactor	1,5-Diamino-2-methylpentane and isophthalic acid	[48,49]
Polyamide-6,6	252.6	55	200	Glass reactor	*para*- and *meta* -xylylenediamine	[50]
Poly(stryrene-*co*-4-vinylbenzaldehyde)	NR	NR	60	Ball-milling process	See Figure 7	[51]
Poly(*L*-lactide) and poly(*D*-lactide)	220	170	180	Twin Screw Mixer	No substrate was used	[52]

NR: Not reported.

### 4.1. Polypropylene

The study conducted by Qiu et al. in 2004 on grafting maleic anhydride onto polypropylene presented several significant benefits. This process, known for its efficiency and eco-friendliness, particularly in the absence of solvents, has been shown to enhance the properties of polypropylene in various ways [40,41]. While the exact study by Qiu et al. (2004) is not directly cited in the available literature, similar studies provide insights into the benefits of this methodology.

A ball-milling technique was utilized to implement this method. The quantities of polypropylene powder varied, ranging from 15 to 50 g [40,41], mixed with different amounts of maleic anhydride. Benzoyl peroxide served as the initiator. This mixture was placed in each milling jar and subjected to a milling process lasting 8 h at a rotational speed of 300 rpm. The process was conducted in a cyclic pattern, consisting of 50 min of milling followed by a 10-min pause. Temperature measurements were taken both at the beginning of each pause step and immediately after the milling process concluded, with recorded temperatures ranging between 75 and 85 °C (Figure 8). In both instances, they reported a high degree of grafting and purity in the final product. The authors repeated the same procedures for grafting maleic anhydride onto polyethylene, achieving a high degree of grafting and purity in the obtained copolymer [40].

Regarding the mechanism of the reaction, it is pretty challenging to study the mechanism of the grafting reaction in SSM by characterizing the copolymer chemical structure. Although, the researchers reported that it is currently under investigation. It is probable that the initial radicals, due to the homolytic scission of benzoyl peroxide (Figure 8), could be combined with maleic anhydride monomers as well as polypropylene molecular chains to induce the reaction. Then, the interaction of radicals could lead to modifications in the molecular structure.

Among methodology advantages, it is possible to highlight: (1) The improved mechanical properties that include better mixing in blends and composites with other polymers and fillers, as well as improved impact resistance and low-temperature brittleness in blends [53]. (2) The process is energy-efficient, particularly important in industrial applications where cost and energy consumption are critical factors. On the other hand, the process of carrying out the modification is quite complex because achieving the optimal degree of grafting requires careful control of various parameters, which can be complex and challenging. Additionally, this methodology requires specialized equipment since the process may require specific equipment for effective grafting, potentially increasing the investment and operational costs. Finally, maintaining consistent quality in the grafting process can be challenging, especially at a larger industrial scale.

Jain et al. (2005) employed SSM for the preparation of polypropylene/silica nanocomposites with varying degrees of adhesion between the filler and matrix [42]. The methodology combines solid-state modification with a sol-gel method to create nanocomposites with varying degrees of adhesion between the filler (silica) and the matrix (polypropylene). The preparation of PP-*g*-silica nanocomposites was conducted using tetraethyl orthosilicate (TEOS). TEOS was seamlessly integrated into the polypropylene (PP) within a double-skinned reactor. This mixture was then subjected to a consistent stirring process for approximately 30 min at a temperature of 60 °C. Following the successful dispersion of TEOS, a carefully composed solution of water and ammonium hydroxide (NH_4_OH) was incrementally introduced into the reactor. The molar ratio of TEOS to water (H_2_O) was maintained at a precise ratio of 1:5, and the NH_4_OH concentration was set at 1 wt%, calculated based on the TEOS quantity. Subsequently, the sol-gel reaction was efficiently facilitated within the reactor, maintaining a constant temperature of 60 °C for a duration of 3 h, accompanied by nonstop stirring. This resulted in the formation of a sol within the PP matrix. The sol then underwent a gelling process at an elevated temperature of 80 °C, sustained over a period of 5 h. Finally, to achieve optimal properties, the gelled product was subjected to a thorough drying process under vacuum conditions at a temperature of 120 °C for 24 h.

Interestingly, the use of solid-state modification and sol-gel reactions can enhance the mechanical and thermal properties of the resulting polypropylene-silica nanocomposites. Moreover, the methodology allows for the adjustment of the degree of adhesion between the silica filler and the polypropylene matrix, enabling customization of the composite properties. In the synthesized nanocomposites, there was a notable absence of any degradation in the polypropylene component. Furthermore, the level of grafting achieved in these nanocomposites was found to be on par with that observed in the melt state, indicating a high degree of consistency and effectiveness in the grafting process. However, the combination of solid-state modification and sol-gel methods can be more complex and challenging to optimize compared to more straightforward composite fabrication methods. Additionally, the complexity and potential scalability issues must be considered when applying this method on an industrial scale.

Finally, these reported methodologies involve combining mechanochemistry with external energy sources, which is critical for controlling local heating and enabling chemical reactions that overcome activation energy barriers unsuitable for conventional mechanochemical syntheses [54].

### 4.2. Poly(butylene terephthalate) (PBT)

PBT has been effectively modified using solid-state modification, a user-friendly technique that has led to various changes in its properties. In the field of SSM, this is the thermoplastic polymer that has been widely reported [17,43,44,45,46,47]. The research groups of C. Koning and S. Muñoz-Guerra show only examples involving poly(butylene terephthalate) (PBT) and biobased monomers, e.g., sugar or fatty acid derivatives [43,44,47]. These studies are focused on the microstructure of the copolyesters obtained and their morphology, emphasizing the influence of structures and compositions on thermal properties and crystallinity [47]. C. Lavilla also studied the comparison of copolymers obtained in SSM with those obtained in conventional polycondensation and showed that these methods appeared to be equivalent [46]. The studies conducted by Lavilla et al. and Gubbels et al. [43,44,45,46,47] employed a similar methodology to carry out the solid-state modification process. These procedures were conducted in a specialized reactor, consisting of a glass tube equipped with a sintered glass plate at its base. Incorporated into this setup was a glass coil designed for heat exchange. As part of the process, nitrogen gas was heated by its passage through this coil before being introduced into the reactor. The flow rate of nitrogen was meticulously regulated using a flow meter. This particular method was consistently applied to PBT/1,4-butanediol (1,4BD), PBT/2,3:4,5-di-O-methylene-galactitol (Manx), PBT/2,3:4,5-di-O-methylene-galactitol (Galx), PBT/2,4:3,5-di-O-methylene-D-glucitol (Glux) and PBT/fatty acid dimer diol (FADD). In a typical experiment, a certain amount of a finely powdered mixture of PBT and a selected reagent was carefully placed onto the sintered glass plate, keeping the reactor’s temperature between 160 and 180 °C. Different catalysts were employed in these works, such as dibutyl tin oxide (DBTO), titanium-based, and even the lack of catalyst. This addition ensured the stability of the powder during the modification process. Although these modifications are typically carried out using batch processes on a small scale, there is great potential for continuous processes to be used for this purpose, such as reactive extrusion, as shown by several recent studies on mechanochemistry [55,56].

Gerbehaye et al. have recently advanced this field by designing a new process that transitions polyester SSM from batch to continuous, using PBT and 1,12-dodecanediol (DDO) as model compounds [17]. Initially, a calorimetric method was developed to investigate the key characteristics of the reaction on a small scale. This helped with the selection of the appropriate catalyst using the differential scanning calorimetry (DSC) technique. In the next step, a qualitative kinetic discussion was conducted to confirm the results obtained from the calorimetric study. The study also shed light on the influence of the reaction time on the molecular and thermal features of the copolymers produced. After optimizing the reaction conditions, they were transferred to a gram-scale batch reactor. Finally, a continuous REx process was tested to minimize the reaction time and assess the shear forces in the SSM framework. The reactive extrusion process was conducted using a 15 cm^3^ twin-screw DSM micro compounder, operating at 190 °C and 40 rpm, with a continuous nitrogen flow to maintain an inert atmosphere. The premixing of polybutylene terephthalate and DDO was achieved using Cryo-Mill, adhering to the previously detailed conditions. Subsequently, DBTO, serving as the most sustainable catalyst, was introduced into the system. The extrusion product was eventually extracted at a higher rate of 100 rpm.

The methodology in this work differs from previous ones by using an industrial machine suitable for continuous processing (Figure 9). Furthermore, REx makes this type of mechanical reaction more industrially viable [55,56]. The primary challenge of this work is rebuilding polymer chains, particularly in terms of molecular weight construction. This was evident since higher times in REx, visual inspection of the material indicated more significant degradation. Therefore, one limitation of this methodology is that longer extrusion times result in more significant molecular weight loss. Initially, DDO acts as a plasticizer aiding the exchange reaction, but prolonged exposure leads to melting and potential loss of crystallinity [17]. REx of polyester with a diol monomer seems feasible, but improved chain building might require an additional vacuum step. Traditional melt polycondensation involves transesterification to form oligomers, followed by polycondensation under high vacuum to remove by-products. Applying a similar vacuum step to SSM might enhance the process. This study presented a new process design for recycling polymeric materials, which could make polymers more sustainable and industrially scalable.

### 4.3. Polyamides

In this kind of thermoplastic polymers, two studies in the field of polymer science focused on modifying polyamides through solid-state methods [49,50]. Jeyakumar et al. studied the modification of polyamide-6,6 (PA-6,6) to prepare a comonomer by using p-Xylylenediaminepara- and m-Xylylenediamine [50]. This process was conducted under an inert atmosphere and initially resulted in a decrease in molecular weight due to chain scission. However, the molecular weight increases again with prolonged treatment as the comonomer is incorporated through postcondensation. Additionally, the solid-state modified copolyamides exhibit higher melting and crystallization temperatures than their melt-synthesized counterparts, indicating a non-random, block-like chemical microstructure. On the other hand, the second study investigates the structural and conformational differences in polyamide 6 (PA6) when modified with Dytek A (a semi-aromatic nylon salt of 1,5-diamino-2-methylpentane) and isophthalic acid (IPA) [48,49]. The results showed no co-crystallization between the Dytek A-isophthalic acid salt and PA6 repeat units (Figure 10). Increasing amounts of the DyI salt in the polymer led to a steady decrease in crystallinity. Also, a transformation from trans to gauche conformers in the polymer chain was observed upon heating.

Regarding the experiment parameters employed to carry out the SSM for both thermoplastic polymers. The experimental setup for the SSM was designed using a similar methodology by using a glass reactor, which included a tube for fluidizing purge gas. This setup utilized a ternary salt mixture as the heating medium, composed of 53 wt% potassium nitrate (KNO_3_), 40 wt% sodium nitrite (NaNO_2_), and 7 wt% sodium nitrate (NaNO_3_). The reactor’s core was a glass tube with an inner atmosphere equipped with a sintered glass filter plate at its base. The process involved heating argon gas, which was achieved by passing it through this coil. For accurate monitoring of the SSM reaction temperature, a thermocouple was inserted directly into the mixture of a thermoplastic matrix (polyamide 6 and polyamide 6,6) and the substrates (Table 1). The reactor operation’s temperature varied according to the polyamide utilized (Table 1).

In the SSP process, nylon salts were initially combined with the amorphous phase of either PA6 or PA-6,6. This combination was achieved through a solution mixing method. Following this, the solvent was evaporated. Subsequently, transamidation reactions were intentionally carried out at temperatures below the melting point (Tm) of the polyamides (Figure 10). Concerning the modification mechanism, the substrates are integrated into polyamides through aminolysis and/or acidolysis processes. During this, the seemingly more reactive diamine cleaves the PA6 and PA-6,6 polymer chains, resulting in an abundance of amine end groups. Subsequently, the monomer attaches to these amine end groups through a condensation reaction.

These studies demonstrate the intricate control of polymer properties through solid-state modification techniques. However, the modification occurred more rapidly in melted polyamides than in SSM in these works. Conversely, the non-random structures in the studies of polyamide 6 and the altered crystallinity for polyamide 6,6 highlight how subtle changes in polymer processing can significantly impact the resulting material’s properties. On the other hand, the scalability of these approaches is limited because they were performed in a glass tube reactor with a batch process.

### 4.4. Poly(stryrene-co-4-vinylbenzaldehyde) or Poly(styrene-co-4-VBA)

**Figure 7:**
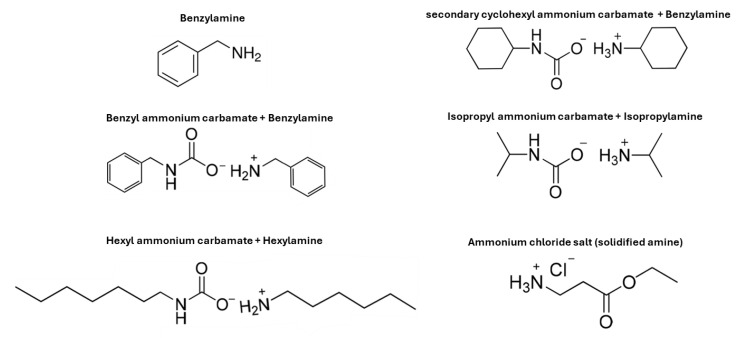
Different comonomers utilized to conduct solid state modification on poly(styrene-*co*-4-vinylbenzaldehyde) where conversion (%) was >99%.

**Figure 8:**
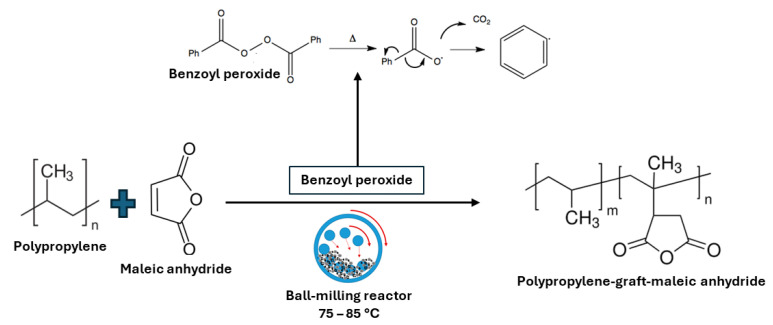
Schematic illustration of modification by SSM (ball-milling) of polypropylene by employing maleic anhydride as the substrate.

**Figure 9:**
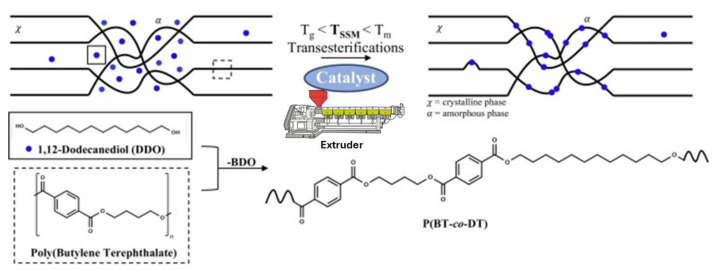
Schematic representation of the SSM by extrusion between PBT and DDO [17].

**Figure 10:**
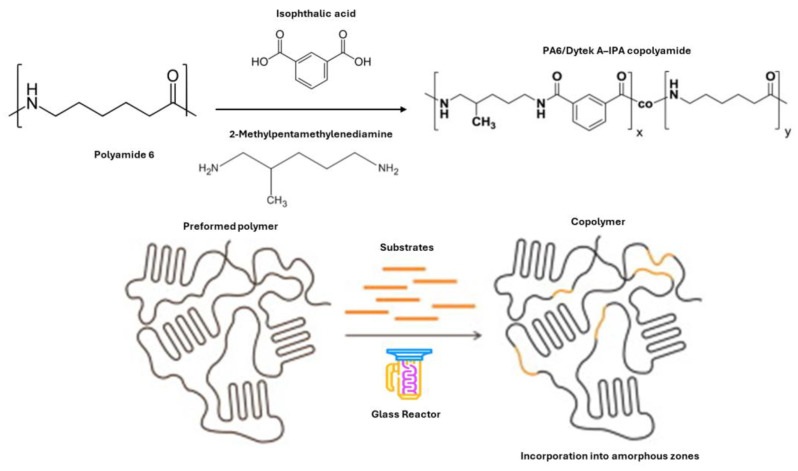
Schematic representation of the SSM between PA6 with Dytek A and IPA.

This work outlines an innovative approach to mechanochemical post-polymerization modification of aldehyde-rich polymers [51]. This method facilitates Schiff base synthesis with various amines (Figure 7) by utilizing a ball-milling technique [51]. The process is characterized by its simplicity, negating the need for solvents or additional reagents aligning with green chemistry principles. This approach influences the well-known and efficient reaction between primary amines and aldehydes or ketones, which typically proceeds rapidly and yields high results with minimal waste production, the only by-product being water.

The experimental procedure involved adding a blend of poly(styrene-co-4-VBA) (0.10 g) and benzyl ammonium carbamate salt (90 mg) to a 10 mL stainless-steel milling container set at a frequency of 30 Hz, which was conducted for 30 min.

The results of this study showcased the effectiveness of a solvent-free, mechanochemical method for post-polymerization modification of poly(styrene-co-4-VBA). The high-speed ball milling process successfully catalyzed the condensation of aldehyde-containing polymers with amine derivatives. The vibrating balls’ vigorous agitation and energy input were critical in achieving polymer modification, with conversion rates exceeding 99% in just 30 min. A significant drawback of this process is its limited scalability, particularly in terms of batch operation.

### 4.5. Poly(L-lactide) (PLLA) and Poly(D-lactide) (PDLA)

The process of stereocomplexation, which involves the interaction between PLLA and PDLA, offers a promising approach to enhance the properties of polylactide (PLA). This enhancement includes increased mechanical strength, thermal stability, and resistance to hydrolysis. Recently, several effective techniques have been developed for creating high-molecular-weight, linear PLLA and PDLA-based polylactide stereocomplexes (sc-PLA). Yet, a significant challenge remains in achieving pure sc-PLA in melt-processed products. This is due to the poor melt stability of sc-PLA, where its ability to re-form sc crystallites after complete melting is markedly reduced. As a result, a mixture of homochiral (hc) and sc crystallites tends to form.

In response to this challenge, we introduce a straightforward method to produce sc-PLA with enhanced melt stability. This method involves melt-blending equimolar amounts of PLLA and PDLA at a low temperature (180 °C), with a small quantity (0.1–0.5 wt%) of a cross-linker. During this process, sc crystallites form quickly. Concurrently, a minor cross-linking occurs among the PLLA and PDLA chains within the amorphous phase, while chains in the crystalline phase remain largely unaffected by the cross-linking reaction. This selective cross-linking in the amorphous phase not only establishes numerous cross-linking points at the ends of the chain couples to prevent complete separation upon melting but also preserves a large portion of long, crystallizable PLA segments within the initially formed sc crystallites. This preservation endows the resulting sc-PLA with remarkable recrystallization capability upon cooling. Remarkably, the formation and reformation of sc crystallites during continuous melting and recrystallization cycles are found to be fully reversible, with no formation of hc crystallites observed.

Conversely, this research paves the way for industrial-scale production of high-performance PLA products using standard melt-processing equipment, a development with significant potential. However, a notable disadvantage is the limited industrial use of these specific PLA stereoisomers, which could pose challenges for widespread adoption.

## 5. Outlooks and Perspective in the Future

Versatility and Customization through SSM: SSM is recognized for its adaptability in modifying thermoplastic polymers. This flexibility is crucial for creating custom-designed materials suitable for various applications, such as high-performance fibers, films, and advanced composites. The future of SSM lies in expanding the range of functionalities that can be incorporated into these polymers, enhancing material properties like strength, flexibility, and heat resistance. For example, SSM can rejuvenate aged polymers in polymer recycling, efficiently integrating them into new products with desirable properties, even in the presence of contaminants.

### 5.1. Eco-Friendly and Cost-Effective

SSM’s eco-friendly and cost-effective nature makes it a valuable method, particularly for high-end recycling and upcycling processes. Future developments may include further reducing environmental impacts and optimizing the process for large-scale industrial applications, focusing on energy-efficient recycling processes. Notably, in the domain of polymer recycling, SSM can be vital in transforming waste polymers into high-value products, thereby contributing to a more sustainable circular economy.

### 5.2. Process Simplification and Scalability

Although SSM processes can be complex and require meticulous control of parameters, ongoing research aims to simplify these processes. Innovations may involve combining mechanochemistry with external energy sources to overcome activation energy barriers, thereby making SSM more accessible and scalable. However, for recycled thermoplastic polymers, this field of research is challenging due to their aging, the presence of additives, fillers, and pollutants, and the fact that some thermoplastic polymers are blends.

### 5.3. Polymer-Specific Innovations

SSM has been used to modify various polymers such as polypropylene, polyethylene, poly(stryrene-*co*-4-vinylbenzaldehyde), poly(butylene terephthalate) (PBT), PLA stereoisomers and polyamides. Future innovations could include more efficient grafting techniques for polypropylene, optimizing continuous processes in PBT and PLA stereoisomers, as well as the scaling up modification processes for polyamides like polypropylene, polyethylene, PA-6,6, PA6 and poly(stryrene-*co*-4-vinylbenzaldehyde). These innovations could revolutionize recovering and repurposing polymers in the recycling sector, turning previously non-recyclable materials into valuable resources. For instance, applying SSM in recycling could convert polymer blends or contaminated polymer wastes into high-quality, application-specific materials, overcoming traditional recycling barriers.

## 6. Conclusions

In terms of the potential for innovation and progress, the solid-state modification of thermoplastic polymers holds significant potential for both innovation and progress. This includes environmental benefits, creating tailored materials, and overcoming process optimization and scalability challenges.

Regarding scaling up for industrial applications, one of the primary challenges for the future is scaling up SSM processes for industrial use. This will require advancements in technology, considerations of economic viability, and maintaining quality control at a larger scale.

Finally, in advancements in recycling and circular economy, the potential of SSM in recycling and enhancing the properties of recycled polymers presents an exciting area for future research. Developing more efficient recycling methods using SSM could contribute significantly to a circular economy in the polymer industry.

## Data Availability

All necessary data to evaluate the study's conclusions are presented in the tables within the main text.
